# The Characteristic of $\{10\bar{1}2\}<10\bar{1}\bar{1}>$ Twin of Ti-10V-2Fe-3Al under Planar Wave Detonation

**DOI:** 10.3390/ma16206739

**Published:** 2023-10-18

**Authors:** Tong Wang, Ping Yang, Jin Zhang, Xin-Fu Gu

**Affiliations:** 1 School of Materials Science and Engineering, University of Science and Technology Beijing, Beijing 100083, China; 18813075238@163.com (T.W.); yangp@mater.ustb.edu.cn (P.Y.); 2 Institute for Advanced Materials and Technology, University of Science and Technology Beijing, Beijing 100083, China; zhangjin@ustb.edu.cn

**Keywords:** Ti alloy, EBSD, twin, planar wave detonation

## Abstract

The microstructure evolution of the twin of TB6 (Ti-10V-2Fe-3Al) under planar wave detonation was studied. The initial microstructure of the alloy consists of an α and β phase. It is found that twin deformation is operated in only the α phase due to the limited slip system in this phase. α grains are mainly rotated from $\{10\bar{1}0\}$ to {0002} during the deformation due to the $\{10\bar{1}2\}<10\bar{1}\bar{1}>$ twin. Twin variant selection is found in this study, and the orientation of all $\{10\bar{1}2\}$ twins is oriented at {0002} in different α grains with different deformation degrees. The twin variant selection is well explained based on the strain relaxation along the loading axis and the Schmid factor for twinning shear.

## 1. Introduction

Titanium and its alloys have drawn more and more attention in various fields be-cause of their multiple characteristics [1], such as low density, excellent corrosion resistance, and high strength. Titanium alloy is usually divided into α alloy, α + β alloy, and β alloy according to the amount of β stabilized elements, where β is the high-temperature phase with body-centered cubic (bcc) structure, while α is the low-temperature phase with close-packed hexagonal structure (hcp) structure. In β alloy, a full β phase can be kept during quenching, but it can be held in α + β two-phase region to obtain a microstructure mixed with α and β phases, and the Burgers orientation relationship, i.e., ${\left\{0001\right\}}_{\alpha }//{\left\{110\right\}}_{\beta }$ and ${<11\bar{2}0>}_{\alpha }//{<1\bar{1}1>}_{\beta }$, is usually held between two phases [2,3,4,5,6]. The volume ratio depends on the processing temperature and holding time. The alloy with dual-phase microstructure is mostly studied because of its superior performance. β can transform to α at a slow cooling rate, but also can transform to α’ or α’’ at a higher cooling rate or deformation strain. TB6 (Ti-10V-2Fe-3Al, Ti1023) is one of the most applicable β titanium alloys. To obtain the most desired mechanical properties, the microstructure could be adjusted through deformation and heat treatment in different periods [7] or via transformations at high temperatures and high pressures [8,9]. β alloys are mostly treated above transus temperature (T_β_) and water-quenched or air-cooling to obtain β solid solution, and α phase could be gained by following annealing [10]. However, martensite is usually produced in rapid cooling such as water-quenched for TB6, which could increase the strength and decrease the toughness of the material, in addition, the martensite could be also produced during cold deformation of β phase titanium alloys [11], while twin is mostly generated in α phase during deformation because of limited slip systems.

Continuous shear along the twin plane and twin direction happens in a specific area to produce twins, and the orientation of the deformed and undeformed grain presents mirror symmetry about the twin plane [12]. The most familiar twin types are tensile and compression twins such as $\{10\bar{1}2\}<10\bar{1}\bar{1}>$ and $\{10\bar{1}\bar{1}\}<10\bar{1}2>$ [13,14,15,16,17]. Pure titanium was a common material of twinning. Xu et al. [18] investigated the microstructure evolution of pure titanium rolling at room temperature, finding that $\{10\bar{1}2\}$ twin was produced during rolling at the intersection of two $\{11\bar{2}1\}$ compression twins. Sun et al. [19] studied the dynamic plastic deformation of pure titanium with fine grains, in which nanoscale $\{11\bar{2}2\}$ compression twin was observed and the texture was affected thereby. In some cases, there was not one type of twin existing in the deformation microstructure. Ye et al. [20] studied the influence of initial texture on twinning, and one variant of $\{10\bar{1}2\}$ tensile twin was found in a single grain. Zeng et al. [21] investigated pure titanium compressed at 400 °C, finding that twin type changed from $\{10\bar{1}2\}$ to $\{10\bar{1}\bar{1}\}$ with more severe deformation. Twins were also produced in titanium alloy. Wu et al. [22] investigated the microstructure change in Ti-2Al-2.5Zr under cold deformation: $\{10\bar{1}2\}<10\bar{1}\bar{1}>$ was the predominant twin to be activated, whose variant selection followed Schmid’s law. Qi et al. [23] also observed $\{10\bar{1}2\}$ and $\{10\bar{1}\bar{1}\}$ twins during hot deformation of Ti6246 and Ti6242 (α + β titanium alloy), which weakened the texture intensity of the initial α colony. Zhu et al. [24] studied the microstructure evolution of near-β titanium alloy (Ti-5.5Mo-7.2Al-4.5Zr-2.6Sn-2.1Cr) after split Hopkinson pressure bar experiment in room temperature, $\{11\bar{2}2\}<11\bar{2}\bar{3}>$ compression twin is found in α grains with a lot of dislocation accumulated in the boundary of the twin and parent phase. Chen et al. [25] also found a twin was produced in the α phase in Ti-6.5Al-Mo-V-2Zr alloy. Xiao et al. [26] studied the deformation mechanisms of β titanium alloy Ti-2Al-9.2Mo-2Fe at the strain rates of 1000 s^−1^: in such high strain rates, the mechanisms are {332} twinning, stress-induced martensitic transformation, stress-induced ω phase, and dislocation slips. However, the efforts aimed to study the β phase change under high strain rates dominate, and there are few investigations on the change in the α phase under high strain rates in α or α + β titanium alloys. Yan et al. [27] studied the deformation mechanisms of Ti6321 with duplex structure at different strain rates from 10^−3^ s^−1^ to 10^5^ s^−1^, revealing that dislocation slip cannot well accommodate the plastic deformation at the strain rate of 10^5^ s^−1^, and $\{12\bar{1}2\}<\bar{1}011>$ tensile twins have been found as a large number of parallel twins.

As one of the most used titanium alloys, the deformation and mechanisms of microstructure evolution in TB6 are complicated. Duan et al. [28] revealed the deformation mechanism of TB6 under hot compression was sensitive to deformation temperature and strain rates, dynamic recovery, and recrystallization were the predominant softening mechanisms. Samiee et al. [29] deformed the TB6 below T_β_ and found stressed-induced martensite after deformation, and Wang et al. [30] observed a similar mechanism in cyclic deformation: the strain rates had obvious effects on the mechanical properties. Qiu et al. [31] conducted the compression tests on the as-cast TB6 titanium alloy at tstrain rates of 0.001–10 s^−1^, concluding that dynamic recovery, dynamic recrystallization, and flow localization behavior were the main mechanism for microstructure change at the strain rates of 0.001, 0.011, and 10 s^−1^, respectively, and the high sensitivity of the strain rate for deforming of TB6 was also found by others [32,33,34,35] for the strain rates from 0.01 s^−1^ to 100 s^−1^. Jackson et al. [36] concluded that the dominant deformation mechanism at high strain rates and high temperature during forging was dislocation slip. Different from low strain rates, deformation mechanisms are unique at high strain rates above 10^3^ s^−1^. Zhang et al. [37] studied the effect of solution temperature on the mechanical properties using split Hopkinson pressure bar at high strain rates, the compression mode is exhibited as the typical shear failure. Zou et al. [38] constructed a Johnson–Cook dynamic constitutive model for high strain rate deformation, the yield stress of TB6 titanium alloy exhibited tension–compression asymmetry, strain rate hardening, and thermal softening effects. Chen et al. [39] investigated the deformation mechanism of TB6 at high strain rates of 2000 s^−1^ and 4000 s^−1^, revealing that net-like and band-like α’’ and internal twin inside the martensite. Yi et al. [40] studied the deformation mechanisms of TB6 at the strain rates of 3000 s^−1^ in different temperatures, adiabatic shear bands were found in all temperatures with different features, the length of which was becoming longer with the increase in temperature. However, Zhang et al. [41] found $\{10\bar{1}2\}$ twins inside α grains after detonation loading, but the characteristic of the generated twin had not been further explored.

In this work, a planar wave denotation was conducted for TB6 alloy to investigate the deformation twin in α grains under a high strain rate. Electron Back-Scattered Diffraction (EBSD) is used to identify crystallographic features of twins, furthermore, the preference of twins in the matrix is rationalized based on the Schmid factors and shear process.

## 2. Materials and Methods

**Figure 1:**
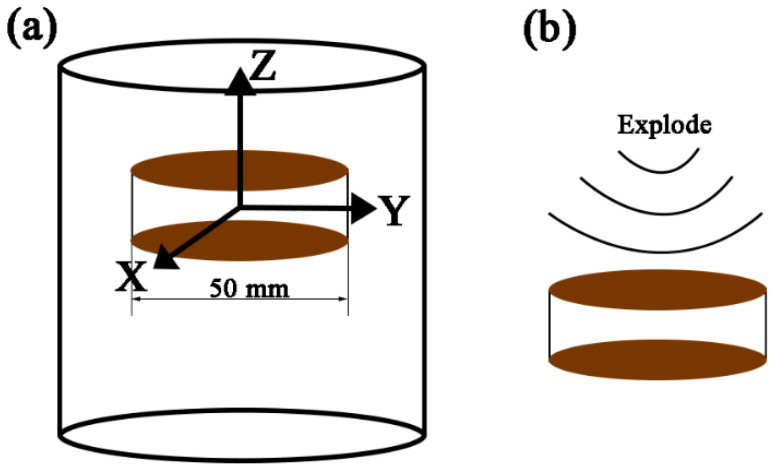
The cutting way of specimen used in planar wave experiment as a dimension of Φ50 × 10 mm. (**a**) The cutting way of plates from the TB6 bar; (**b**) The planar wave denotation bearing on the plates.

The material used in this experiment was cut from a forged TB6 bar (Ti-10V-2Fe-3Al), and the specimens for planar wave detonation were cut from TB6 bar with the dimension of Φ50 mm × 5 mm, as shown in Figure 1. The specimens were stacked one by one and loaded by detonation, which was deformed in different degrees from near to far from the explosion center, in addition, from center to edge of the specimen, the strain rate is about 10^5^–10^6^ s^−1^. In this work, low, middle, and high deformation specimens were selected to investigate the twinning characteristics under various deformations, which are denoted by LD, MD, and HD (with deformation strain about 10%, 40%, and 60%), respectively. The samples with a dimension of 8 mm × 5 mm × 5 mm (along with X × Y × Z) were cut from different deformation locations of specimens. The X-Z plane paralleling to the forging axis (Z) was used to show the microstructure evolution after deformation along the Z axis (loading direction), and the IPF-Z image was used to reveal the texture because it is perpendicular to the loading direction. The surface was mechanically ground and then electrochemical polishing was conducted to remove the surface stress with the voltage and current of 27.8 V and about 300 mA, respectively. Finally, the sample was etched by the Kroll agent to reveal the microstructure.

Scanning electron microscopy (SEM) was adopted to research the microstructure change because of the fine grain which could not be observed in an optical microscope. As SEM fittings, EBSD was used to study the grain orientation change before and after deformation. In the meantime, the EBSD data were processed using Aztec crystal software (Aztec crystal 2.1) to obtain the twin relationship, the volume fraction of α and β, etc. The tolerance angle for determining the twin boundaries in the EBSD mapping is set to be 10°.

## 3. Results

### 3.1. Initial Microstructure

**Figure 2:**
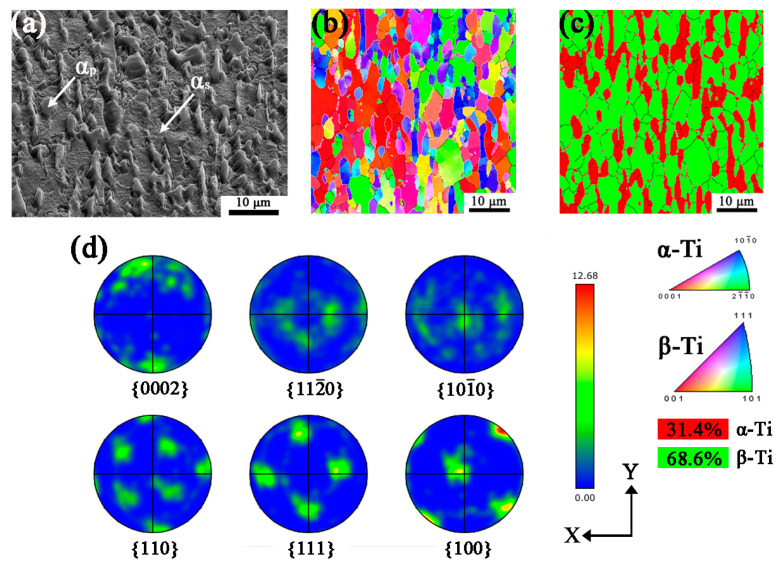
The initial microstructure and texture of raw TB6. (**a**) SEM image; (**b**) Orientation mapping with IPF-Z; (**c**) Phase map; (**d**) Pole figures.

Figure 2 shows the initial microstructure and orientation of raw materials. From Figure 2a, it is found that TB6 is composed of lamellar primary α(α_p_), acicular secondary α(α_s_), and β matrix, α_s_ are separated because TB6 raw bar was forged in the two-phase area and α_s_ formed during cooling of the forged alloy. Although α_s_ can be observed in SEM images, it could not be observed in EBSD with the scanning step of 0.2 μm, such as in Figure 2b,c, since the grain size of α_s_ is very fine. In Figure 2b,c, the black and white lines represent the grain boundaries and phase boundaries, respectively. In addition, the red and green colors in the phase mapping Figure 2c correspond to α and β phase, respectively. Compared with Figure 2b,c, the orientation of α and β is mainly $\{10\bar{1}0\}$//X-Y plane in α phase, while {110} and {100}//X-Y plane in β phase, respectively. The volume fraction of α and β is 31.4% and 68.6%, respectively. Figure 2d shows the pole figures of α and β of {0002}, $\{10\bar{1}0\}$, and $\{11\bar{2}0\}$ and {100}, {101}, and {111}, respectively. The texture type of α is $\{10\bar{1}0\}$ and β is mainly {110} and {100} texture with a maximum intensity of 12.68. It should be noted that the texture intensity of α is lower than β, corresponding to the results of Figure 2d, two of which follow the Burgers orientation relationship with ${\left\{0001\right\}}_{\alpha }//{\left\{110\right\}}_{\beta }$, ${<11\bar{2}0>}_{\alpha }//{<1\bar{1}1>}_{\beta }$.

### 3.2. Microstructure of LD Specimen

**Figure 3:**
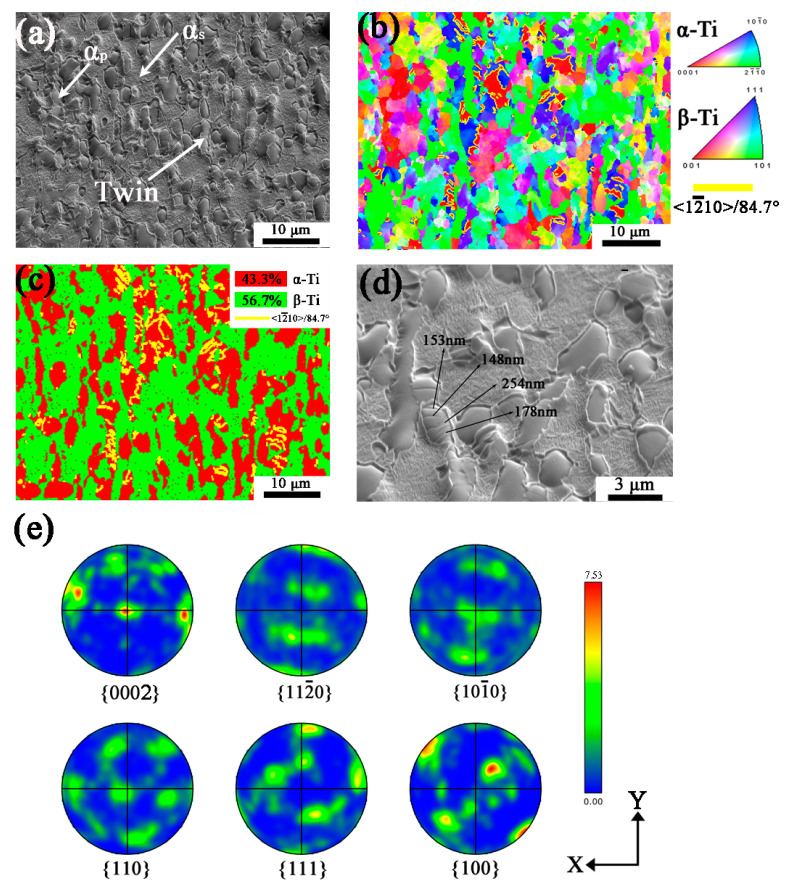
The sample of low deformation (LD) after planar wave detonation. (**a**) SEM image; (**b**) Orientation mapping with IPF-Z; (**c**) Phase map; (**d**) SEM image (High magnification); (**e**) Pole figures. Yellow lines in (**b**–**d**) show twin boundaries.

Figure 3a–e shows the microstructure and orientation of TB6 in low deformation (LD) conditions. In Figure 3a, α and β are elongated along the Z axis, in addition, the twin is observed in the sample inside α_p_ because of insufficient slip systems of α during deformation, where the yellow lines in Figure 3b,c represent twin boundaries of $<1\bar{2}10>/84.7{}^{\circ}$, the orientation of α_p_ is mostly $\{10\bar{1}0\}$//X-Y plane, with few {0002} and $\{11\bar{2}0\}$//X-Y plane. Little grain misorientation variation (2–5°) inside α grain without twin, while the misorientation of twinned grains is about 10°. The volume fraction of β phase is about 56.7% according to the phase mapping in Figure 3c. The orientation of the parallel twin is almost oriented {0002}//X-Y plane and generated in $\{10\bar{1}0\}$ grains, which represents the preferred growth of the twin in low deformation conditions. To evaluate the size of the twins, the high magnification SEM image in Figure 3d shows the width of twins from 150 nm to 250 nm. Figure 3e shows that the α orientation of LD specimen is {0002} and $\{10\bar{1}0\}$, corresponding to twin and α_p_ grain, respectively. By comparing the pole figures of α and β phase, two phases also follow the Burgers orientation relationship, and the texture intensity of α is increased compared with the α phase in the undeformed state in Figure 2d.

### 3.3. Microstructure of MD Specimen

**Figure 4:**
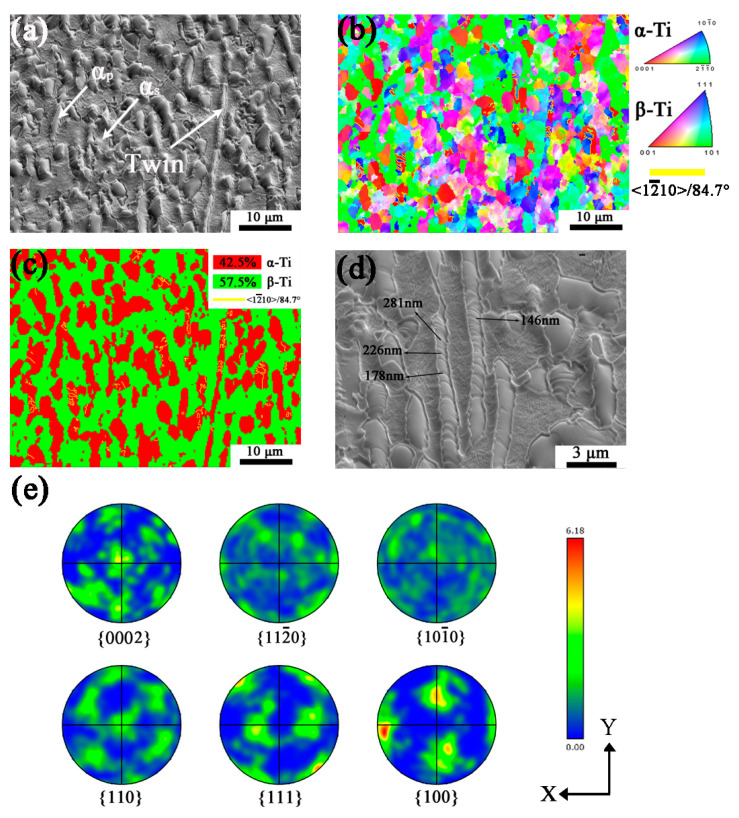
The sample of middle deformation (MD) after planar wave detonation. (**a**) SEM image; (**b**) Orientation mapping with IPF-Z; (**c**) Phase map; (**d**) SEM image (High magnification); (**e**) Pole figures. Yellow lines in (**b**–**d**) show twin boundaries.

The microstructure and orientation of the MD specimen are shown in Figure 4. Twins are also found in the SEM image of Figure 4a. In Figure 4b,c, α is mostly {0002} and $\{10\bar{1}0\}$//X-Y with few $\{11\bar{2}0\}$ based on the orientation colors, while β is mostly {110}//X-Y plane. As for the MD specimen, the misorientation of twinned grains is about 15°, greater than the LD specimen, representing more intense deformation of α grains. The phase fraction of α and β is 42.5% and 57.5%, respectively, according to the phase mapping in Figure 4c. Twins are mostly generated in $\{10\bar{1}0\}$ grains with few existing in $\{11\bar{2}0\}$ grains, the orientation of which is {0002}//X-Y plane, and the twin boundaries of $<1\bar{2}10>/84.7{}^{\circ}$ are shown in yellow lines in Figure 4b–c. Figure 4d shows the high-magnification SEM image of MD specimen, the twins are parallel to each other and more than LD specimen, whose width is from 150 nm to 300 nm. As represented in Figure 4e, the texture of α is {0002} and weak $\{10\bar{1}0\}$, in the meantime, β is mostly {110}//X-Y plane. It shows the orientation of α changes from $\{10\bar{1}0\}$ to {0002} with larger deformation.

### 3.4. Microstructure of HD Specimen

**Figure 5:**
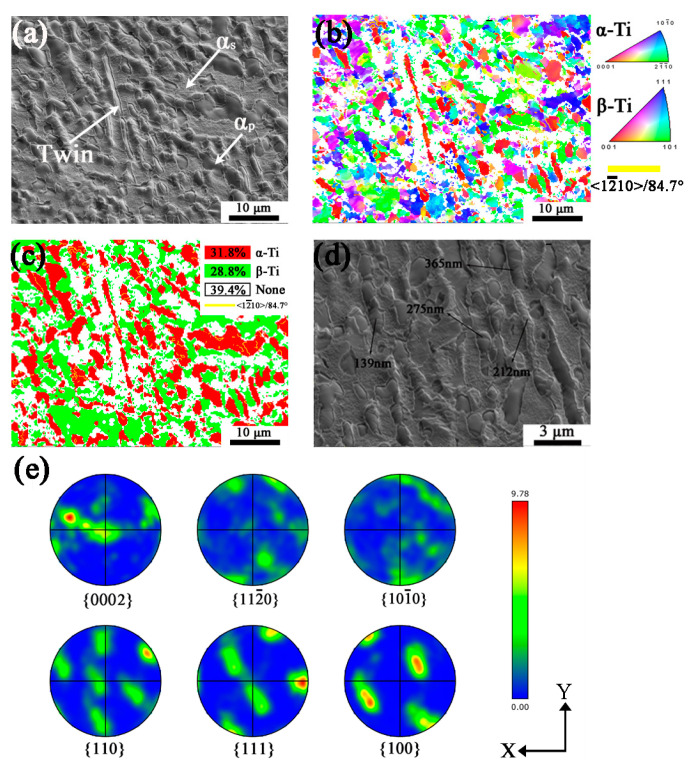
The sample of high deformation (HD) after planar wave detonation. (**a**) SEM image; (**b**) Orientation mapping with IPF-Z; (**c**) Phase map; (**d**) SEM image (High magnification); (**e**) Pole figures. Yellow lines in (**b**–**d**) show twin boundaries.

Figure 5a–e shows the microstructure and orientation mapping of the HD specimen after the planar wave detonation. As shown in Figure 5a, the surface of the elongated α grains is found more rougher, and more strip grains (twins) are found. Zero solution up to 39.4% in EBSD mapping is presented in Figure 5b,c, because of heavier deformation where the Kikuchi patterns cannot be well captured. Based on Figure 5b,c, the orientation of α is mostly {0002} with few $\{10\bar{1}0\}$ and $\{11\bar{2}0\}$ parallel to the X-Y plane, and β is mainly oriented with {110}//X-Y plane. It should be noted that some parallel internal twin existing in the SEM picture is not resolvable in orientation mapping with EBSD, which is may also due to the deformation defects in the grains. To calculate the width of twins in HD specimen, high magnification of SEM image shows the severely deformed grains including twins, the width of most twins is about 250 nm, and there are few below 200 nm and above 300 nm. The orientations of twins are multiple in {0002} grain, which will be discussed in detail later. From Figure 5e, the texture of α is close to {0002} and the β matrix orientation is also with {110}//X-Y plane, as the same as the case in the lower deformed case.

## 4. Discussion

### 4.1. Twin Types

**Figure 6:**
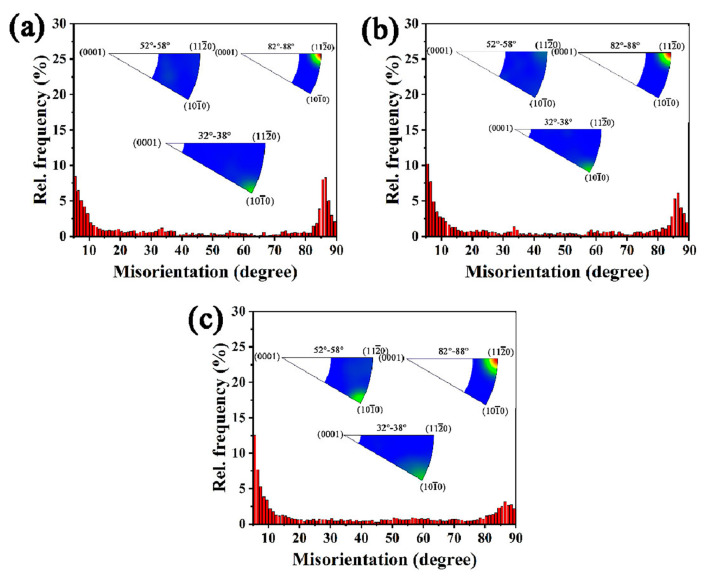
Misorientation angle distribution of α phase after planar wave detonation. (**a**) LD; (**b**) MD; (**c**) HD.

To investigate the twin type operated during deformation, the misorientation angle distribution of the α phase after the planar wave detonation is shown in Figure 6. It is revealed that the misorientation angle is concentrated at both the angles less than 10° and the angles more than 80°, and the former is due to low-angle boundaries formed in deformation, while the latter is due to the twinning process. In addition, when the deformation strain increases from LD to HD, the relative frequency of the misorientation angles less than 10° increases, while that more than 80° decreases gradually, but the distribution peaks become broader. The peak misorientation angle/axis between 82° and 88° is concentrated in $<11\bar{2}0>$ axis, which is belonging to $<1\bar{2}10>/84.7{}^{\circ}$ twin mentioned in Figure 3b, Figure 4b and Figure 5b. Moreover, the peak misorientation angle/axis between 32° and 38° is concentrated in $\{10\bar{1}0\}$ axis as shown in the inner Figure in Figure 6a–c, which may belong to $<\bar{1}100>/35.1{}^{\circ}$ twins. The misorientation angle/axis between 52° and 58° is only discovered in the HD specimen with the misorientation axis around $<10\bar{1}0>$ directions. Since the high angle boundaries other than the angles around 84.7° exhibit a low area fraction, then $<1\bar{2}10>/84.7{}^{\circ}$ twin is discussed in detail.

### 4.2. $<1\bar{2}10>/84.7{}^{\circ}$($\{10\bar{1}2\}<10\bar{1}\bar{1}>$) Twins in LD Specimen

**Figure 7:**
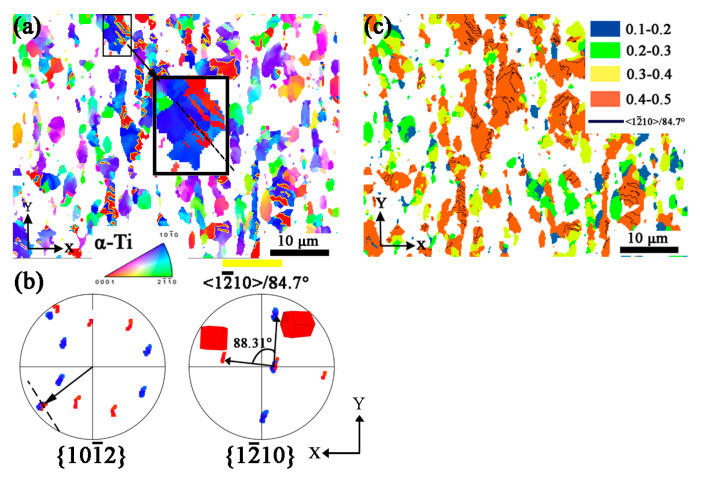
EBSD results of α phase in LD specimen. (**a**) Orientation mapping with IPF-Z; (**b**) Pole figures of $\{10\bar{1}2\}$ and $\{1\bar{2}10\}$ of black square area in (**a**); (**c**) The Schmid factor mapping of $\{10\bar{1}2\}<10\bar{1}\bar{1}>$ twinning system of LD specimen (Blue color represents the Schmid factor of 0.1–0.2, green color represents the Schmid factor of 0.2–0.3, yellow color represents the Schmid factor of 0.3–0.4, red color represents the Schmid factor of 0.4–0.5, black line represents the twin boundary of $<1\bar{2}10>/84.7{}^{\circ}$).

Figure 7a shows the orientation mapping of only α phase and twins inside it, and the yellow line represents $<1\bar{2}10>/84.7{}^{\circ}$ twin boundaries. It shows only {0002}//X-Y plane twin (in red color) produced in $\{10\bar{1}0\}$ grain. The $\{10\bar{1}2\}$ and $\{11\bar{2}0\}$ pole figures for the area marked by a black rectangle are shown in Figure 7b. Apart from the misorientation mentioned before, the pole figures directly show the twin relationship, i.e., one of the $\{10\bar{1}2\}$ or $\{11\bar{2}0\}$ poles coincide with each other, respectively. Moreover, the trace of the twin boundary (black line) is normal to the coincidence $\{10\bar{1}2\}$ poles, and it further supports the proposed twin type. The misorientation angles around 84.7° are found in Figure 6, and the scattering distribution is due to the deformation of α phase. The variation of the orientations can be also found in the pole figures in Figure 7b, for example, the rotation angle of twin around $\{1\bar{2}10\}$ for 88.31° is schematically shown.

The high Schmid factor grains are preferred to be twinned according to the research of HCP metal Mg and Zr [42]. The Schmid factor mapping for $\{10\bar{1}2\}<10\bar{1}\bar{1}>$ twinning system of LD specimen is given in Figure 7c, with the loading direction parallel to Z. The twins are observed in grains with a Schmid factor of 0.4–0.5, which represents the $\{10\bar{1}2\}<10\bar{1}\bar{1}>$ tensile twins produced in the grains with high Schmid factor, other than grains of 0.1–0.4. The variants of twins are determined by high Schmid factors. In the meantime, the Schmid factor of twins is also high, with a similar color in the matrix.

### 4.3. $<1\bar{2}10>/84.7{}^{\circ}$($\{10\bar{1}2\}<10\bar{1}\bar{1}>$) Twins in MD Specimen

**Figure 8:**
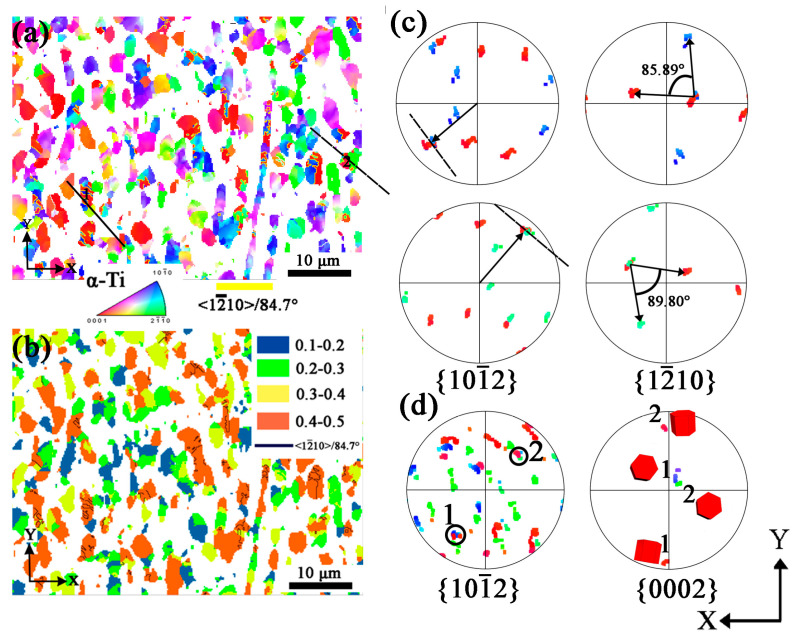
EBSD results of α phase in MD specimen. (**a**) Orientation mapping with IPF-Z; (**b**) The Schmid factor mapping of $\{10\bar{1}2\}<10\bar{1}\bar{1}>$ twinning system (Blue color represents the Schmid factor of 0.1–0.2, green color represents the Schmid factor of 0.2–0.3, yellow color represents the Schmid factor of 0.3–0.4, red color represents the Schmid factor of 0.4–0.5, black line represents the twin boundary of $<1\bar{2}10>/84.7{}^{\circ}$); (**c**) Pole figures of $\{10\bar{1}2\}$ and $\{1\bar{2}10\}$ of $<1\bar{2}10>/84.7{}^{\circ}$ twin of area-1 and area-2; (**d**) Pole figures of $\{10\bar{1}2\}$ and {0002} of area-1 and area-2.

Different from LD specimen, MD specimen is deformed more severely. The orientation mapping is shown in Figure 8a. The $<1\bar{2}10>/84.7{}^{\circ}$ twins are oriented at {0002}, with red color in Figure 8a, and most twins are in $\{10\bar{1}0\}$ grains and few in $\{11\bar{2}0\}$ grains. Typical examples are marked as area-1 and area-2 in Figure 8a and selected for further analysis. Figure 8d shows $\{10\bar{1}2\}$ and {0002} pole figures: the $\{10\bar{1}2\}$ poles are superimposed and the {0002} poles are about 90° apart and these are typical features of $<1\bar{2}10>/84.7{}^{\circ}$ twins, showing twins growing in both $\{10\bar{1}0\}$ and $\{11\bar{2}0\}$ grains during planar wave detonation. The $\{10\bar{1}2\}<10\bar{1}\bar{1}>$ tensile twin is produced because the X and Y direction is stretched, while the Z direction is compressed during planar wave detonation. The c-axis of red and green grains is compressed and stretched, corresponding to the red point and blue (green) points, respectively, in {0002} pole figure in Figure 8d, and the red grains are twins while the blue and green grains are matrix in Figure 8a. The $\{10\bar{1}2\}$ and $\{11\bar{2}0\}$ pole figures of area-1 and area-2 are shown in Figure 8c. Again, the twin trace agrees well with the superimposed poles in the $\{10\bar{1}2\}$ pole figure. Due to the deformation, the angles of misorientation between the twin and the matrix vary from 85.89° to 89.80° around $<11\bar{2}0>,$ as shown in Figure 8c of red-blue and red-green conincident points, respectively.

Figure 8b shows the Schmid factor mapping of $\{10\bar{1}2\}<10\bar{1}\bar{1}>$ twinning system of MD specimen. Like the LD specimen, $\{10\bar{1}2\}<10\bar{1}\bar{1}>$ tensile twins are found in grains with Schmid factor 0.4–0.5. The elongated grains exhibit a high Schmid factor of 0.4–0.5, in which twins are found in these grains.

### 4.4. $<1\bar{2}10>/84.7{}^{\circ}$($\{10\bar{1}2\}<10\bar{1}\bar{1}>$) Twins in HD Specimen

**Figure 9:**
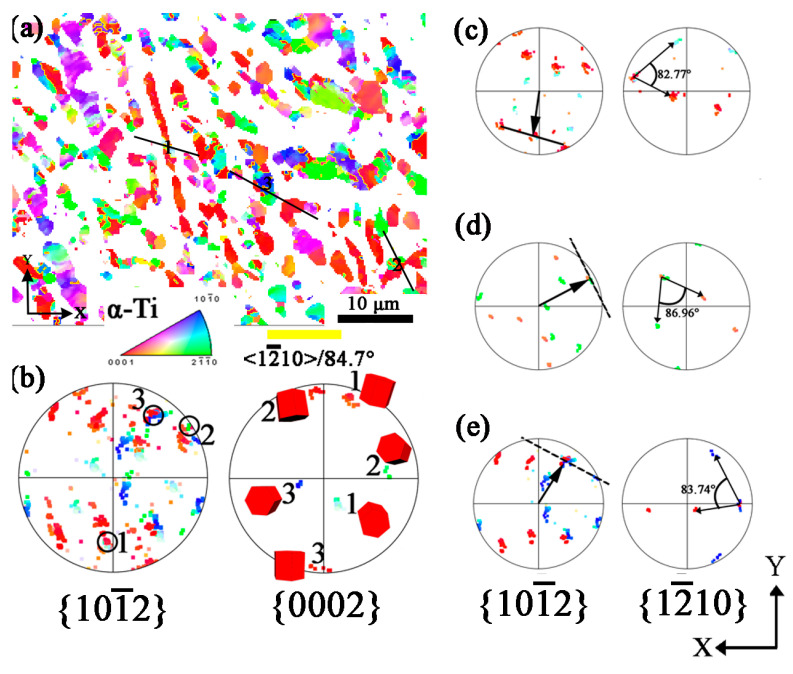
EBSD results of α phase in HD specimen. (**a**) Orientation mapping with IPF-Z; (**b**) Pole figures of $\{10\bar{1}2\}$ and {0002} of area-1, area-2, and area-3; (**c**) area-1; (**d**) area-2; (**e**) area-3.

**Figure 10:**
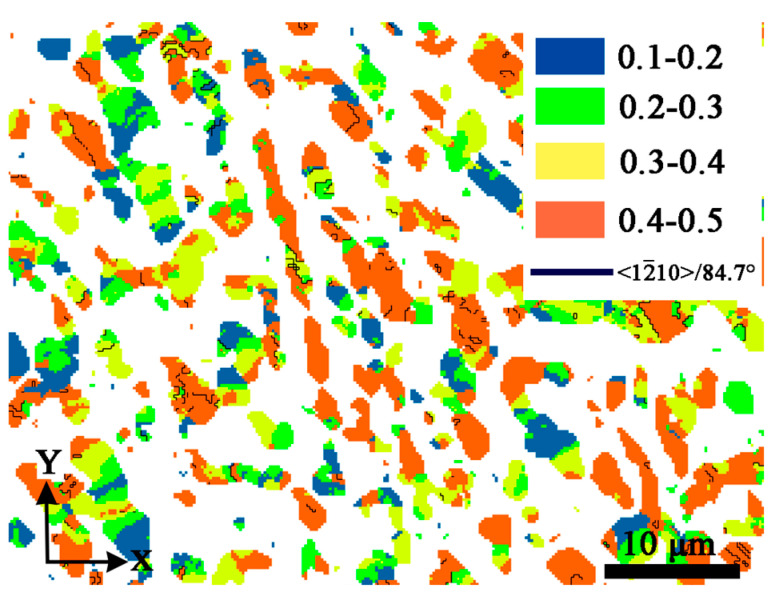
The Schmid factor mapping of $\{10\bar{1}2\}<10\bar{1}\bar{1}>$ twinning system of HD specimen (Blue color represents the Schmid factor of 0.1–0.2, green color represents the Schmid factor of 0.2–0.3, yellow color represents the Schmid factor of 0.3–0.4, red color represents the Schmid factor of 0.4–0.5, black line represents the twin boundary of $<1\bar{2}10>/84.7{}^{\circ}$).

The HD specimen suffers more severe deformation as shown by zero solution in the EBSD pattern, as mentioned previously. The orientation mapping is shown in Figure 9. The twin is also oriented with {0002}, with the red color in the mapping. According to the $\{10\bar{1}2\}$ pole figure in Figure 9b, in which the superimposed gray-red (1), green-red (2), and blue-red (3) points prove twins producing in $\{10\bar{1}0\}$, $\{11\bar{2}0\}$ and other-oriented grains. Figure 9c–e shows the $\{10\bar{1}2\}$ and $\{11\bar{2}0\}$ pole figures at points 1, 2, and 3, and the twin trace agrees well with the superimposed $\{10\bar{1}2\}$ poles in Figure 9c–e. Similarly, the misorientation between the matrix and twins varies, and the angles of 82.77°, 86.96°, and 83.74° are shown in positions 1, 2, and 3, respectively. Figure 10 shows the Schmid factor mapping of $\{10\bar{1}2\}<10\bar{1}\bar{1}>$ twinning system of HD specimen, the twins are produced in grains of Schmid factor above 0.3, rather than only in high Schmid factor, proving the twins are easy to produce in heavy deformation conditions. Twinning is also activated in low Schmid factor grains in some research, such as in pure titanium using X-ray diffraction, in which $\{10\bar{1}2\}$ tensile twinning was formed because of the influence of adjacent grains in low Schmid factor grains [43].

### 4.5. Twinning Preferentially on Account of Grain Size

**Figure 11:**
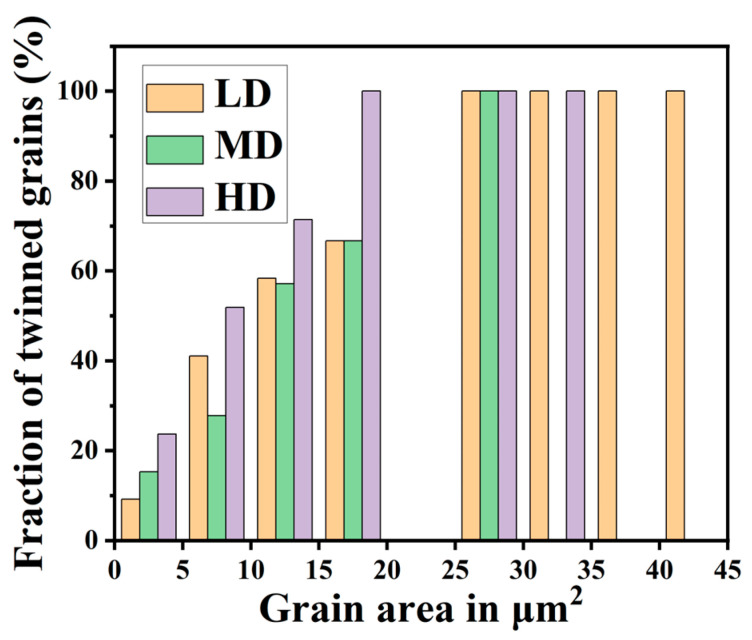
Fraction of twinned grains (=number of twinned α grains/total number of α grains in the size range) in different deformation specimens. (Yellow column represents LD specimen, green column represents MD specimen, purple column represents HD specimen).

The global Schmid factor may not be the only reason for the twin variant selection since the variations of grain size and shape may cause local stress to be different from macroscopic stress, and these characteristics of grains should be considered during twinning. Figure 11 shows the fraction of twinned grains (equals to the number of twinned α grains/total number of α grains of different area grains) in different area α phases of different deformation specimens. The propensity of twin activation increases with increasing grain size in all different deformation specimens; however, twinning is not found in the grain size of 20–25 μm^2^ in all specimens. In addition, the influence of grain size on twinning propensity is obvious in smaller sizes, a similar effect was also found in pure Mg [44]. Arul Kumar et al. [45] also found twinning restrained around small grains and facilitated around large grains. In this work, the adjacent grains of smaller grains are hard constitutions of α_s_+β, high twin back-stress restrains the creation of twins. While α_s_ could be ignored around large grains, twinning is promoted during twin tip impinging on a larger adjacent grain.

### 4.6. Twin Variant Selection

**Figure 12:**
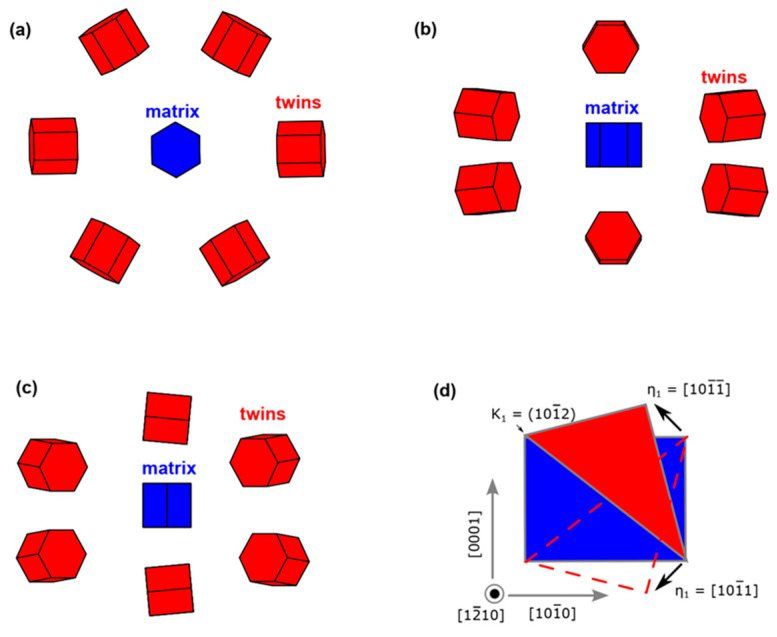
Six variants of $\{10\bar{1}2\}$ twins viewed from (**a**) <0001>; (**b**) $<10\bar{1}0>$; (**c**) $<11\bar{2}0>$ directions in the matrix. The matrix is shown in blue while the twin variants are in red; (**d**) schematic diagram of the twinning process.

The six twin variants with the matrix are shown in Figure 12a–c from different view directions in the matrix. The twin variants are due to symmetries of the matrix, thus crystallographic equivalent twin orientations exist [46]. In Figure 12a, the variants are viewed along the [0001] direction in the matrix, the [0001] direction exhibits six-fold rotational symmetries [47], and six twin variants in red colors are generated. In our experiment, only several particular variants are presented, which is called variant selection. The operated twin variants correspond to the variants with the high Schmid factors found in our experiment, and this is also easy to understand by the illustrations from the view of the shearing process. In the $<10\bar{1}0>$ viewed variants, the twin variants can orient in different directions in Figure 12b, and the {0002} plane in variants nearly parallel to $\{10\bar{1}0\}$ in the matrix is observed. In Figure 12b, this corresponds to the upper and lower variants, and the misorientation between these two variants is about 10.6°, which may be another reason for the scattering of twin orientations. Figure 12d is a schematic diagram of the shearing process for generating a $\{10\bar{1}2\}$ twin along the shear direction $<10\bar{1}\bar{1}>$. The tensile along the [0001] direction or compressing along the $<10\bar{1}0>$ grains will facilitate the twin process, while opposite stress will prohibit the twin process. Therefore, the compression along $<10\bar{1}0>$ grains in the experiment will generate two twin variants as shown in Figure 12d by shear along $[10\bar{1}\bar{1}]$ and $[10\bar{1}1]$ directions due to the compression strain along $[10\bar{1}0]$, and the {0002} in twin will be oriented close to the $\{10\bar{1}0\}$ in the matrix, consistent with our experimental observations. Our result shows that the twin variant selection can be rationalized by the Schmid factor even under explosive loading.

Local Schmid factor and Luster–Morris parameter (m’) are the important factors to evaluate the twinning nucleation, which represent how the twinning orientation adapts the stress concentration by dislocation accumulation. Previous studies found twin variants with a low Schmid factor may have high m’. Wang et al. [43] reported that high m’ could promote the twin variant of low Schmid factor grains. Guan et al. [48] explained the twin variant selection in low Schmid factor grains occurred because of slip transmission from neighboring grains. However, the twinned grains in this work are preferred in high Schmid factor grains, and twins within grains with low Schmid factor are possible with larger deformation strain.

## 5. Conclusions

In this work, the characteristic of deformation after planar wave detonation is studied by the EBSD method, and the main conclusions are as follows,

(1) After the planar wave denotation, $<1\bar{2}10>/84.7{}^{\circ}$ type twins are observed in α grains, mainly in $\{10\bar{1}0\}$ grains.
(2) The orientation of α changes from $\{10\bar{1}0\}$ to {0002} and the maximum intensity of the $\{10\bar{1}0\}$ poles decrease due to the twinning process.
(3) As the deformation increases, the deviation from ideal twin misorientation becomes large and causes a wider distribution of the twin misorientation. The twin from other-oriented grains will begin to operate.
(4) The twin variant selection during the detonation is rationalized by considering the twinning shear process, and the twin variants with high Schmid factors should be studied in future.

## Data Availability

The data presented in this study are available on request from the corresponding author.
